# Therapeutic Effect of Human iPS-Cell–Derived Myeloid Cells Expressing IFN-β against Peritoneally Disseminated Cancer in Xenograft Models

**DOI:** 10.1371/journal.pone.0067567

**Published:** 2013-06-24

**Authors:** Chihiro Koba, Miwa Haruta, Yusuke Matsunaga, Keiko Matsumura, Eriko Haga, Yuko Sasaki, Tokunori Ikeda, Koutaro Takamatsu, Yasuharu Nishimura, Satoru Senju

**Affiliations:** 1 Department of Immunogenetics, Graduate School of Medical Sciences, Kumamoto University, Kumamoto, Japan; 2 CREST, Japan Science and Technology Agency, Kawaguchi, Japan; University of Pécs Medical School, Hungary

## Abstract

We recently developed a method to generate myeloid cells with proliferation capacity from human iPS cells. iPS-ML (iPS-cell–derived myeloid/macrophage line), generated by introducing proliferation and anti-senescence factors into iPS-cell–derived myeloid cells, grew continuously in an M-CSF–dependent manner. A large number of cells exhibiting macrophage-like properties can be readily obtained by using this technology. In the current study, we evaluated the possible application of iPS-ML in anti-cancer therapy. We established a model of peritoneally disseminated gastric cancer by intraperitoneally injecting NUGC-4 human gastric cancer cells into SCID mice. When iPS-ML were injected intraperitoneally into the mice with pre-established peritoneal NUGC-4 tumors, iPS-ML massively accumulated and infiltrated into the tumor tissues. iPS-ML expressing IFN-β (iPS-ML/IFN-β) significantly inhibited the intra-peritoneal growth of NUGC-4 cancer. Furthermore, iPS-ML/IFN-β also inhibited the growth of human pancreatic cancer MIAPaCa-2 in a similar model. iPS-ML are therefore a promising treatment agent for peritoneally disseminated cancers, for which no standard treatment is currently available.

## Introduction

Macrophages play essential roles to maintain homeostasis in the body. They reside in all tissues in the body and are engaged in various functions, such as eliminating invading pathogens, remodeling tissues, and clearing dead cells. Additionally, macrophage infiltration is frequently observed in various cancers [1]. Recent studies indicate that these tumor-associated macrophages (TAM) mainly promote progression of cancer by accelerating the local invasion and metastasis of cancers [2]. In contrast, other studies demonstrate tumoricidal effect of macrophages [3], [4]. Based on the anti-cancer effects of macrophages observed in pre-clinical studies, application of macrophages to cancer therapy has been tried; for example, transfer of macrophages pre-activated with IFN-γ was tested as a potential treatment agent for cancer patients [5]–[9]. However, no clear therapeutic benefit against cancer has been observed thus far in the macrophage therapy.

To establish macrophage therapy as a more effective anti-cancer therapy, improving the method for supplying macrophages is necessary. In the reported clinical trials, macrophages used for therapeutic purpose were generated from donor peripheral blood monocytes that were isolated by leukapheresis. However, peripheral blood monocytes isolated from cannot be readily propagated. The number of macrophages generated by such methods is therefore limited (at most 10^9^ to 10^10^), and may be insufficient to achieve clinical effects. If sufficient numbers (for example, more than 10^10^) of macrophages with the potent anti-cancer property could be repeatedly administered, we could realize effective anti-cancer therapy with macrophages.

Pluripotent stem cells, such as embryonic stem (ES) cells or induced pluripotent stem (iPS) cells, can propagate indefinitely and possess the ability to differentiate into various types of somatic cells, including blood cells. Destruction of a human embryo is necessary to generate human ES cells. iPS cells, on the other hand, can be generated by introducing several defined factors into somatic cells derived from any donor [10]–[13]. Thus, iPS cell technology can overcome ethical issues as well as the histoincompatibility issue between the therapeutic donor cells and the recipient, and future application of iPS cells to clinical medicine is expected [14], [15].

Several groups, including ours, have thus far established methods to generate macrophages from mouse or human pluripotent stem cells [16]–[24]. However, human pluripotent stemα cells yield lower number of macrophages than mouse pluripotent stem cells. So far established methods generate human macrophage numbers that are less than 100 times the number of the undifferentiated iPS cells used as the starting materials; in addition, generating macrophages by conventional methods takes more than one month. Thus, conventional methods are too laborious and expensive to be applied to practical medicine.

Recently, we established a method to induce proliferation of the iPS-cell–derived myeloid cells (iPS-MC) by lentivirus-mediated transduction of genes that can promote cell proliferation or inhibit cell senescence, such as cMYC plus BMI1, EZH2, or MDM2, to generate an iPS-cell–derived myeloid/macrophage cell line (iPS-ML) [25]. iPS-ML can proliferate in an M-CSF-dependent manner for at least several months while retaining the potential to differentiate into dendritic cells (iPS-ML-DC) with a potent T cell-stimulating capacity.

In the current study, we evaluated the potential of using iPS-ML as anti-cancer effector cells. We investigated whether or not genetically modified iPS-ML expressing anti-HER2 antibody or interferon (IFN) could exert therapeutic effect against peritoneally disseminated gastric and pancreatic cancers in xenograft models.

## Materials and Methods

### Cells and reagents

This study was approved by ethics review board of Kumamoto University Graduate School of Medical Sciences. A human gastric cancer cell line, NUGC-4, and a human pancreatic cancer cell line, MIAPaCa-2, were provided by the Japanese Collection of Research Bioresources (JCRB, Osaka, Japan). Methods for the generation, maintenance, and genetic modification of human iPS cells have been described previously [21].

### Flow cytometric analysis

The following mAbs conjugated with FITC or PE were purchased from BD Pharmingen (San Diego, CA), Beckman Coulter (Brea, CA), Miltenyi Biotec (Bergish-Gladbach, Germany), Sigma (St. Louis, MO), or eBioscience (San Diego, CA): anti-CD45 (clone HI30, mouse IgG1), anti-CD33 (WM53, mouse IgG1), anti-CD36 (FA6.152, mouse IgG1), anti-CD11b (ICRF44, mouse IgG1), anti-CD14 (61D3, mouse IgG1), anti-CD4 (11830, mouse IgG2a), anti-CD97 (VIM3b, mouse IgG1), anti-CD13 (WM15, mouse IgG1), anti-CD87 (62022, mouse IgG1), anti-CD115 (2-3A3-1B10, rat IgG2a), anti-CD116 (4H1, mouse IgG1), anti-TLR2 (T2.5, mouse IgG1), anti-TLR4 (HTA125, mouse IgG2a), anti-HER2/neu (Neu 24.7, mouse IgG1), and anti-cMYC (9E10, mouse IgG1). Isotype-matched controls, mouse IgG2a (G155-178) and mouse IgG1 (MOPC-21), were used. The cell samples were treated with FcR-blocking reagent (Miltenyi Biotec) for 10 min, stained with the fluorochrome-conjugated mAbs for 30 min, and washed 3 times with PBS/FCS (2%). Stained cell samples were analyzed using a FACScan (Becton Dickinson) flow cytometer.

### Zymosan phagocytosis assay

Cell suspensions in DMEM/10% FCS were added to 48-well culture plates (2×10^5^ cells/well in 200 µL) and incubated at 37°C for 2 hours to allow the cells to adhere to the plates. FITC-labeled zymosan A particles (Molecular Probes) were added to the wells (2×10^6^ particles/well in 200 µL). After incubation for the indicated period, cells were microscopically observed (Axio Observer Z1, Carl Zeiss, Oberkochen, Germany) and harvested by trypsin/EDTA treatment for flow cytometric analysis using a FACScan flow cytometer.

### Construction of expression vectors

A plasmid clone containing cDNA for a single chain variable region fragment (scFv) specific to human HER2/neu was a gift of Lieberman (Addgene plasmid #10794) [26]. The scFv sequence was linked by PCR to nucleotide sequences for a cMYC tag (EQKLISEEDL) and a C-terminal fragment of mouse FcγRI. The protein-coding DNA fragment was first cloned into pENTR-D-Topo (Life Technologies) and subsequently transferred into a mammalian expression vector pCAG-IRES-Puro, which is driven by the CAG promoter and includes an internal ribosomal entry site (IRES)-puromycin *N*-acetyl-transferase gene cassette. cDNAs for human IFN-α, IFN-β, IFN-γ, TNF-α, TRAIL, and FAS-ligand were provided by NITE Biological Resource Center (Kazusa, Japan), Kazusa DNA Research Center (Kazusa, Japan), or RIKEN BRC (Tsukuba, Japan). A lentivirus vector, CSII-EF, and packaging constructs were generated by H. Miyoshi (RIKEN BRC) and colleagues [27]. The cDNA fragments were inserted into the CSII-EF plasmid along with IRES-hygromycin phosphotransferase to generate lentiviral expression constructs. Recombinant lentivirus was produced and purified by a previously described method [21].

### Generation of iPS-ML expressing scFv

A plasmid vector (pCAG-IRES-Puro) encoding anti-HER2 scFv was introduced into human iPS cells by electroporation and selected using puromycin (5 µg/mL). Stably transfected clones were isolated by a previously described method [21]. Subsequently, iPS cell clones carrying the anti-HER2 scFv construct were placed into differentiation culture to generate iPS-MC/anti-HER2. The iPS-MC expressing scFv specific to HER2 were transduced with lentivirus vectors for cMYC plus BMI1, or cMYC plus EZH2, to generate iPS-ML. The method for generating and maintaining iPS-ML has been previously reported [25].

### Genetic modification of iPS-ML/scFv to express additional factors

iPS-ML were transduced with lentivirus vector encoding IFN-α, IFN-β, IFN-γ, TNF-α, FAS-ligand, or TRAIL. To select cells stably expressing the transgenes, the cells were cultured in a medium containing hygromycin (0.5∼2 mg/mL). To quantitate the production of transgene-derived cytokines and FAS-ligand, the transfected iPS-ML were cultured (1×10^5^ cells/well in 200 µL) in 96-well flat-bottomed culture plates for 24 hours, and the concentration of cytokines and FAS-ligand in the culture supernatant was measured by using ELISA kits purchased from Endogen or R&D Systems. TRAIL expression was examined by flow cytometric analysis.

### Analysis of cancer cell sensitivity to cytokines

NUGC-4 or MIAPaCa-2 cells were cultured (4×10^5^ cells/well in 1 mL) in 24-well culture plates in the presence or absence of 10 ng/mL recombinant IFN-α, IFN-β, IFN-γ, or TNF-α for 24 hours. Subsequently, the cells were recovered and stained with FITC-labeled Annexin-V (Biovision, Mountain View, CA) and analyzed on a FACScan flow cytometer. Luciferase-expressing cancer cells were cultured (5×10^3^ cells/well in 200 µL) in 96-well culture plates (B&W Isoplate, Wallac) in the presence of 10 ng/mL recombinant IFN-α, IFN-β, IFN-γ, or TNF-α. Three days later, luciferase substrate solution (SteadyLite Plus, Perkin-Elmer) was added (50 µL/well), and luminescence was measured on a micro-plate reader (TriStar, BertholdTech, Bad Wildbad, Germany).

### Analysis of anti-tumor activity of iPS-ML *in vitro*

NUGC-4 cells (5×10^3^ cells/well) expressing luciferase were cultured with or without iPS-ML (2.5×10^4^ cells/well) in 96-well flat-bottomed culture plates (B&W Isoplate, Perkin-Elmer). After a 3-day culture, 50 µL/well of luciferase substrate solution was added, and luminescence was measured on a micro-plate reader.

### Analysis of iPS-ML infiltration into cancer tissues in SCID mice

Mouse experiments were approved by the animal research committee of Kumamoto University. Green fluorescence protein (GFP)-expressing NUGC-4 cells (5×10^6^ cells/mouse) were injected into the peritoneal cavity of SCID mice. After 15 days, iPS-ML were labeled with PKH26 (Sigma), following the manufacturer’s instructions, and were intraperitoneally (i.p.) injected into the mice (3×10^6^ cells/mouse). Mice were sacrificed the following day and subjected to fluorescence analysis to macroscopically detect the location of NUGC-4 tumors and iPS-ML on a NightOWL II (Berthold Technologies, Bad Wildbad, Germany). NUGC-4/GFP was detected with 475 nm excitation and 520 nm emission filters, and PKH26-labeled iPS-ML was detected with 550 nm excitation and 600 nm emission filters. For microscopic examination, cancer tissues in the greater omentum were removed, fixed in 4% paraformaldehyde/PBS, and embedded in Tissue-TEK OCT compound (Sakura Finetechnical, Tokyo, Japan). Tissue sections of 20-μm thickness were made on a cryostat and analyzed on a fluorescence microscope (Axio Observer Z1, Carl Zeiss, Oberkochen, Germany).

### Analysis of anti-tumor activity of iPS-ML *in vivo*

SCID mice were i.p. injected with the cancer cells (5×10^6^ cells/mouse). On day 3 or 4, the mice were subjected to luminescence image analysis to examine tumor establishment. Subsequently, mice with established tumors were randomly divided into treatment and control groups. Mice in the treatment group were injected with iPS-ML according to the indicated schedule, and cancer cell growth was monitored in mice by luminescence imaging analysis. The magnitude of cancer growth was determined by the change of total luminescence counts for each mouse.

## Results

### Characterization of human iPS-cell–derived proliferating myeloid cells

**Figure 1:**
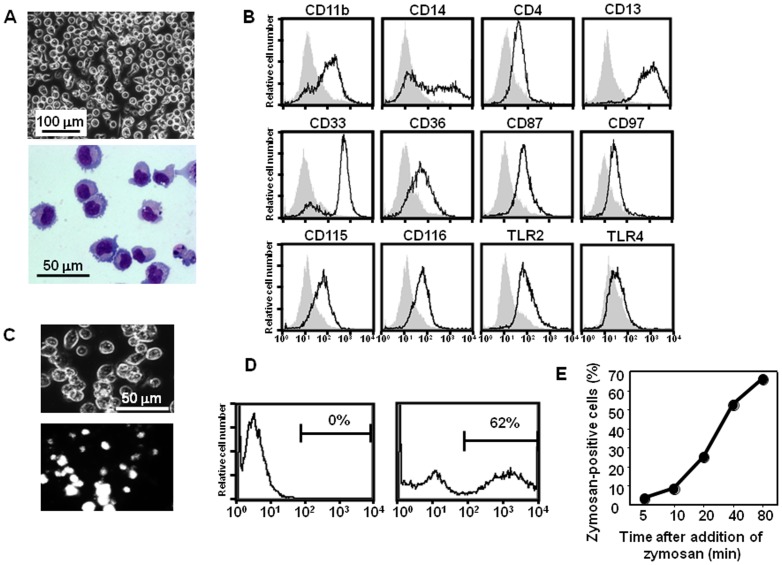
Characterization of iPS-ML as macrophages. **A.** A phase-contrast image of live iPS-ML in a culture plate (upper) and an image of iPS-ML stained with May-Giemsa on a slide glass (lower) are shown. **B.** Cell-surface expression of macrophage marker molecules CD11b, CD14, CD4, CD13, CD33, CD36, CD87, CD97, CD115, CD116, TLR2, and TLR4 on iPS-ML was analyzed by flow cytometry. The staining profiles of the specific mAb (thick lines) and an isotype-matched control mAb (grey area) are shown. **C.** iPS-ML in culture plates were added with FITC-labeled zymosan particles. Phase-contrast (upper) and fluorescence (lower) images after a 90-min incubation are shown. **D.** After a 40-min incubation in the presence or absence of zymosans, cells were harvested using trypsin/EDTA and then analyzed on a flow cytometer. Percentages of cells with high fluorescence intensity indicating intracellular zymosan are shown. **E.** Time course for phagocytosis is shown. Data shown are mean ± SD of duplicate assays.

We previously established a procedure to generate myelomonocytic cells with proliferating capacity (iPS-ML) by lentivirus-mediated transduction of cMYC plus BMI-1 into human iPS cell-derived myeloid cells (iPS-MC) [25]. iPS-ML grew mostly in suspension in an M-CSF–dependent manner. They expressed several macrophage makers, and were heterogeneous in the morphology and in the expression of some of the cell surface molecules (Fig. 1A, B).

To analyze the phagocytic ability of iPS-ML, we microscopically observed the iPS-ML culture after adding FITC-labeled zymosan particles. After a 90-min incubation, fluorescence signals were detected in most cells, indicating that most of the iPS-ML ingested zymosan particles (Fig. 1C). Approximately 60% of the iPS-ML contained zymosan particles after 40 min incubation with FITC-labeled zymosan particles, as assessed by flow cytometric analysis (Fig. 1D). A time course for phagocytosis is shown in Figure 1E.

### Anti-cancer activity of iPS-ML expressing anti-HER2 scFv *in vitro*

**Figure 2:**
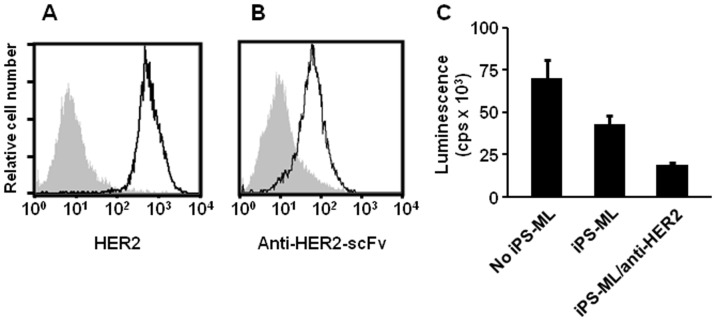
Effect of iPS-ML/anti-HER2 against HER2-expressing NUGC-4 gastric cancer cells *in vitro*. **A.** HER2/neu expression on NUGC-4 human gastric cancer cells was analyzed. The staining profiles of anti-HER2 mAb (thick line) and an isotype-matched control antibody (grey area) are shown. **B**. Cell-surface expression of anti-HER2 scFv on iPS-ML (iPS-ML/anti-HER2) was detected by staining with an anti-cMYC-tag antibody. **C.** Luciferase-expressing NUGC-4 cells (5×10^3^ cells/well) were cultured alone or co-cultured in a 96-well culture plate with iPS-ML (1×10^4^ cells/well) with or without anti-HER2 scFv expression. The number of live NUGC-4 cells was measured by luciferase activity after 3-day culture. The data are indicated as the mean + SD of duplicate assays.

A cancer-related antigen, HER2/neu, is expressed by various kinds of human cancers, including breast and gastric cancers [28]. We decided to examine the anti-cancer effect of iPS-ML expressing anti-HER2 scFv against a HER2-expressing gastric cancer cell line, NUGC-4 (Fig. 2A). For this purpose, we generated iPS-ML stably expressing anti-HER2 scFv (iPS-ML/anti-HER2) (Fig. 2B). iPS-ML/anti-HER2 were generated from iPS-MC derived from an iPS cell clone introduced with an expression vector for anti-HER2 scFv by a previously described method [21]. We sporadically examined and confirmed the expression of the scFv by iPS-ML/anti-HER2.

At first we evaluated the effect of iPS-ML/anti-HER2 against NUGC-4 cells *in vitro*. Firefly luciferase-introduced NUGC-4 cells were co-cultured with iPS-ML with or without anti-HER2 scFv expression. We observed that iPS-ML reduced live NUGC-4 cells, and that expression of anti-HER2 scFv in iPS-ML enhanced the inhibitory effect against the growth of NUGC-4 cells (Fig. 2C).

### Accumulation and infiltration of i.p. injected iPS-ML in tumor tissues

We wanted to evaluate whether iPS-ML had a therapeutic effect on peritoneally disseminated cancer. Macrophage infiltration is frequently observed in clinical samples of cancer tissue [1]. We examined whether or not i.p. administered iPS-ML infiltrated into cancer tissues pre-established in the peritoneal cavity of mice.

To this end, GFP-expressing NUGC-4 human gastric cancer cells, established from a peritoneal metastatic lesion of a diffuse-type gastric cancer patient, were injected i.p. into SCID mice. After 15 days, iPS-ML labeled with red fluorescent dye PKH26 were injected. We simultaneously injected recombinant tissue plasminogen activator (tPA) into the mouse peritoneal cavity, expecting that tPA promoted the infiltration of iPS-ML into tumor tissues. Mice were sacrificed on the following day, and dissected to determine the location of the injected iPS-ML by fluorescence analysis.

**Figure 3:**
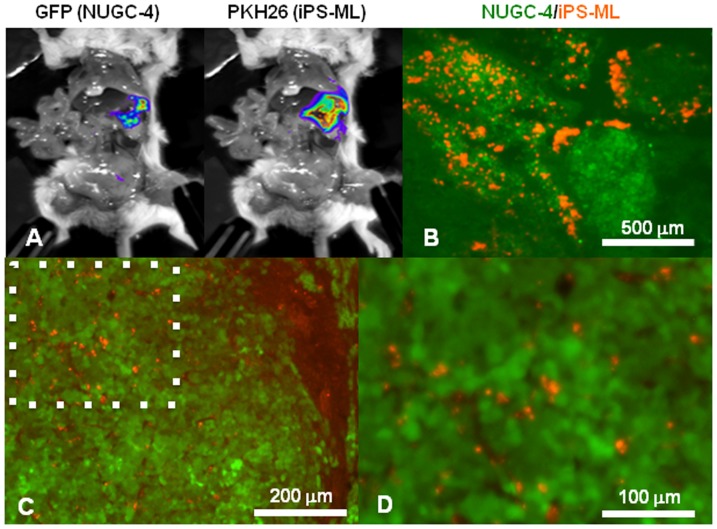
Accumulation and infiltration of iPS-ML in pre-established tumor tissues in mouse peritoneal cavity. **A.** GFP-expressing NUGC-4 cells (5 ×10^6^ cells/mouse) were injected into the peritoneal cavity of SCID mice. After 15 days, iPS-ML labeled with fluorescent PKH26 were injected i.p. into the mice (3×10^6^ cells/mouse). The mice were sacrificed the following day and subjected to fluorescence imaging analysis to determine the location of the NUGC-4-GFP tumors (excitation/emission: 475/520 nm) and PKH26-iPS-ML (excitation/emission: 550/600 nm). **B.** Tumor tissues in the greater omentum of the mice were isolated, and 20-µm thick frozen sections were made. The sections were analyzed on a fluorescence microscope, and a merged image with green fluorescence indicating NUGC-4/GFP cells and red fluorescence indicating PKH26-stained iPS-ML is shown. **C, D** Tissue sections were made by a similar procedure as for **B**, except that tPA was not used. A higher magnification view of the region surrounded by a dotted square in **C** is shown in **D**.

Macroscopic fluorescence analysis detecting GFP (excitation/emission: 475/520 nm) indicated that NUGC-4 tumors mainly localized in the greater omentum (Fig. 3A). Injected iPS-ML detected by PKH26 fluorescence (excitation/emission: 550/600 nm) were also mostly localized in the greater omentum, demonstrating that iPS-ML efficiently accumulated into the tumor tissues. Such a clear accumulation of iPS-ML into the greater omentum was not observed when iPS-ML were inoculated into the mice without established tumors (data not shown).

We then isolated and microscopically examined the tumor tissues. In the tissue section shown in Figure 3B, PKH26-labeled iPS-ML infiltrated into the nest of GFP-expressing NUGC-4 cells. Similar experiments were done without tPA injection, and higher magnification analysis of the tissue sections clearly shows the infiltration of iPS-ML into cancer tissue (Fig. 3C, D). These results indicate that iPS-ML efficiently infiltrated into the cancer tissues, when i.p. injected into mice carrying cancers established in the peritoneal cavity.

### No anti-cancer activity of iPS-ML expressing anti-HER2 scFv *in vivo*

We next examined the effect of iPS-ML/anti-HER2 against NUGC-4 *in vivo*. Luciferase-expressing NUGC-4 cells were inoculated into the peritoneal cavity of SCID mice (5×10^6^ cells/mouse). After 3 days, cancer cell engraftment in the mice was examined by bioluminescence analysis, and mice bearing cancer cells were randomly divided into treatment or control groups. From days 4 to 8, the treatment group mice were injected daily with iPS-ML/anti-HER2 (2×10^7^ cells/mouse). On day 10, the mice were subjected to bioluminescence analysis again to examine the progression of the cancer.

As shown in Figure S1, NUGC-4 tumors in the iPS-ML-treated mice grew even faster than in the control mice. They rather enhanced NUGC-4 cancer cell growth in this *in vivo* model, although statistically non-significant. This may be because iPS-ML/anti-HER2 were affected by the cancer microenvironment to acquire a pro-cancer phenotype.

### Sensitivity of NUGC-4 cells to cytokines and cell-killing molecules

To make iPS-ML able to overcome the cancer microenvironment and to exert anti-cancer effects *in vivo*, we determined to further modify iPS-ML/anti-HER2 to express additional molecules. Cytokines, such as IFNs, are known to induce death or inhibit growth of cancer cells [29], [30]. In addition, these cytokines are known to enhance the anti-cancer activity of macrophages [31]–[33].

We analyzed the sensitivity of NUGC-4 cells to recombinant IFN-α, IFN-β, IFN-γ, or TNF-α. After a 24-hour incubation in the presence either of these factors (10 ng/mL), we analyzed apoptosis by staining the cells with FITC-labeled annexin-V. We observed that all tested cytokines induced certain levels of NUGC-4 cell apoptosis (Fig. S2A). To examine the effect to reduce the number of live NUGC-4 cells, luciferase-expressing NUGC-4 cells were cultured for 3 days in the presence of these factors. Consistent with the annexin-V-staining data, all tested cytokines significantly decreased the number of live NUGC-4 cells (Fig. S2B). In both assays, IFN-β and IFN-γ exhibited the most profound effect.

### Generation of iPS-ML/anti-HER2 expressing additional molecules

We generated lentivirus expression vectors for the IFNs and TNF-α, and introduced them into iPS-ML/anti-HER2. In addition, we introduced lentivirus expression vectors for “apoptosis-inducing factors”, FAS-ligand or TRAIL. We were able to generate transfected iPS-ML that produced cytokines at more than 3 ng/24 hour/10^6^ cells, except for IFN-γ (Fig. S3A). We could generate transfectant iPS-ML producing only a low level of IFN-γ, probably because of toxicity of IFN-γ to iPS-ML. Cell surface expression of TRAIL in the transfected iPS-ML was confirmed by flow cytometric analysis (Fig. S3B).

**Figure 4:**
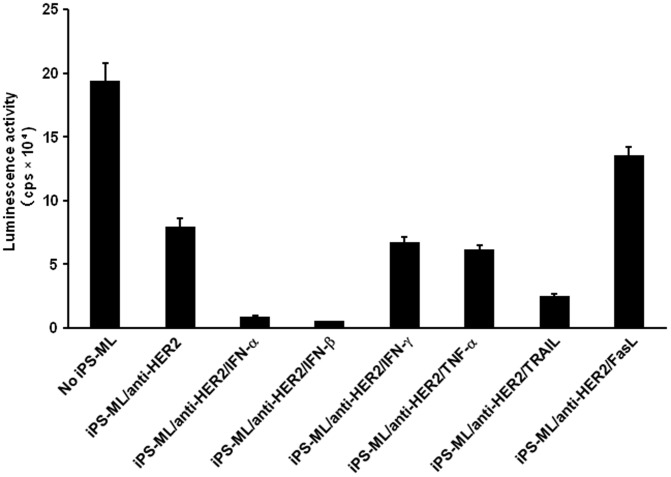
Effect of iPS-ML/anti-HER2 expressing additional factors against NUGC-4 ***in vitro*** **.** Luciferase-expressing NUGC-4 cells were cultured in a 96-well culture plate (5×10^3^ cells/well) with iPS-ML/anti-HER2 expressing IFN-α, IFN-β, IFN-γ, TNF-α, FAS-ligand, or TRAIL (2.5×10^4^ cells/well). The number of live NUGC-4 cells was measured by luminescence analysis after 3-day culture. The data are indicated as the mean + SD of triplicate assays.

We co-cultured the iPS-ML/anti-HER2 expressing additional anti-cancer molecules with luciferase-expressing NUGC-4 cells and analyzed the number of live NUGC-4 cells based on luciferase activity after 3 days (Fig. 4). iPS-ML/anti-HER2 expressing IFN-α, IFN-β, or TRAIL showed a more profound effect to reduce NUGC-4 cells than iPS-ML/anti-HER2, and those expressing IFN-β were the most potent. iPS-ML/anti-HER2 expressing IFN-γ or TNF-α exhibited an effect similar to iPS-ML/anti-HER2. The lack of significant enhancement of the anti-NUGC-4 effect by IFN-γ transduction may be due to that the amount of IFN-γ produced by iPS-ML/anti-HER2/IFN-γ was lower than the level to exert anti-cancer effect. In this experiment, forced expression of FAS-ligand unexpectedly weakened the anti-NUGC-4 effect of iPS-ML/anti-HER2.

### Therapeutic effect of iPS-ML/IFN-β on peritoneally disseminated NUGC-4 gastric cancer cells in SCID mice

Based on the results of *in vitro* experiments, we examined the *in vivo* anti-NUGC-4 effect of iPS-ML/anti-HER2 expressing either IFN-α, IFN-β, or TRAIL. Treatment with neither iPS-ML/anti-HER2/IFN-α nor iPS-ML/anti-HER2/TRAIL showed clear inhibitory effect on the cancer cell growth *in vivo* (data not shown). On the other hand, iPS-ML expressing IFN-β exhibited significant effect to inhibit the growth of the cancer as described below.

**Figure 5:**
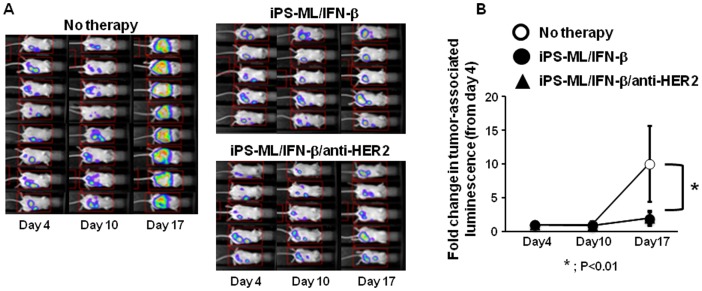
Effect of iPS-ML producing IFN-β with or without anti-HER2 treatment to inhibit the growth of peritoneally disseminated NUGC-4 cells. Luciferase–expressing NUGC-4 cells were injected i.p. into SCID mice (5×10^6^ cells/mouse). On day 4, the mice were subjected to the luminescence imaging analysis. Mice were injected on days 4, 6, 8, 11, 13, and 15 with iPS-ML/IFN-β or iPS-ML/IFN-β/anti-HER2 (2×10^7^ cells/mouse for each injection, n = 5 for each group). As a control, 8 mice were left untreated. All mice were subjected to bioluminescence analysis on days 10 and 17. A. The luminescence images are shown. B. For each mouse, the luminescence signal was calculated as a relative value, where the photon count on day 4 was defined as 1. The mean ± SD of fold-change from day 4 in control and treatment groups are shown.

In the experiments shown in Figure 5, growth of NUGC-4 tumors was monitored by bioluminescence analysis on days 4, 10 and 17 after the cancer cell inoculation. Mice bearing NUGC-4 tumors on day 4 were divided into therapy or no-therapy (control) group. Mice of the therapy groups were injected i.p. with iPS-ML/IFN-β or iPS-ML/anti-HER2/IFN-βfrom day 4 (2×10^7^ cells/injection/mouse, 3 injections per week). Figure 5A shows the image data of the luminescence analysis of the mice. Figure 5B indicates the fold change of luminescence activity from day 4 of the control and treatment groups, demonstrating that tumor growth was inhibited by treatment with iPS-ML/IFN-β or iPS-ML/anti-HER2/IFN-β. iPS-ML/IFN-β and iPS -ML/anti-HER2/IFN-β were equivalently effective, indicating that expression of anti-HER2 is dispensable for anti-cancer effect of iPS-ML producing IFN-β.

Figure S4 shows the results of similar experiments to comparatively examine the effects of iPS-ML, iPS-ML/IFN-β, iPS-ML/anti-HER2, and recombinant IFN-β against NUGC-4 cancer *in vivo*. In consistent with the data shown in Figure 5, treatment with iPS-ML/IFN-β significantly suppressed the progression of cancer. On the other hand, both iPS-ML and iPS-ML/anti-HER2 rather promoted the growth of cancer, although statistically nonsignificant. Injection of 400 ng but not 200 ng/mouse/injection of recombinant IFN-β on the same schedule as iPS-ML injection exhibited some inhibitory effect on the tumor growth, although the effect was not statistically significant.

Collectively, simple iPS-ML did not exhibit anti-cancer effect *in vivo*. Genetic modification to produce IFN-β conferred significant anti-cancer activity to iPS-ML. On the other hand, expression of anti-HER2 scFv did not have such effect.

### Therapeutic effect of iPS-ML/IFN-β against pancreatic cancer in a xenograft model

We next examined the effect of iPS-ML/IFN-β treatment against pancreatic cancer cells. Addition of recombinant IFN-β to MIAPaCa-2 human pancreatic cancer cells induced apoptosis and reduced the number of live cells in *in vitro* experiments (Fig. S5).

**Figure 6:**
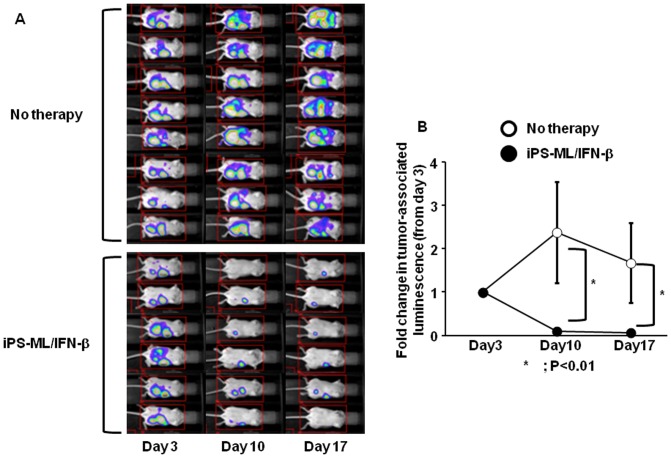
Inhibition of MIAPaCa-2 pancreatic cancer cell growth by iPS-ML/IFN-β. Luciferase–expressing MIAPaCa-2 cells were inoculated i.p. into SCID mice (5×10^6^ cells/mouse), and the mice were subjected to luminescence imaging analysis on day 3. Mice engrafted with cancer cells were randomly divided into treatment (n = 6) and control (n = 8) groups. Mice in the treatment group were injected with iPS-ML/IFN-β (2×10^7^ cells/mouse for each injection) on days 4, 6, 8, 11, 13, and 15. All mice were subjected to bioluminescence analysis on days 10 and 17. The luminescence images are shown in **A**. For each mouse, the change of luminescence signal per mouse was calculated as a relative value, where the photon count on day 3 was defined as 1. The mean ± SD of the fold change in control and treatment groups are shown in **B**.

We examined the effect of iPS-ML/IFN-β against MIAPaCa-2 cells *in vivo*. We could establish a peritoneal cancer model by i.p. injection of the MIAPaCa-2 cells expressing luciferase into SCID mice. In the experiments shown in Figure 6, 6 mice were treated with iPS-ML/IFN-β injection 3 times per week for 2 weeks from day 4; the results of the 8 control mice without treatment are also shown. We observed that iPS-ML/IFN-β treatment significantly inhibited MIAPaCa-2 tumor growth as compared with control mice. The decrease in the average luminescence count from day 10 to day 17 of the control (no therapy) group should have been due to the increase of ascites caused by the cancer, because we observed progressive enlargement of the abdomen in the mice of this group. The results shown in Figure 6 suggest that the therapy with iPS-ML/IFN-β is effective against pancreatic cancer.

## Discussion

Macrophage infiltration is frequently observed in clinical samples of solid cancers, and these macrophages are called TAM. In the present study, we observed that i.p. injected iPS-ML efficiently accumulated and infiltrated into pre-established cancer tissues in the peritoneal cavity of SCID mice (Fig. 3). To examine the tissue infiltration of intravenously administered iPS-ML, we injected PKH26-labeled iPS-ML via tail vein into the mice carrying cancer in the peritoneal cavity. However, we could not detect evident accumulation of iPS-ML in the cancer tissue in this experiment. In the mice, i.v. injected iPS-ML should have distributed systemically, and we could inject at most 2×10^6^ of iPS-ML into the tail vein. Thus, i.v. injectable iPS-ML were not sufficient to be detected in the intra-peritoneal cancer tissues by macroscopic or histological analysis.

Intending to confer anti-cancer activity to iPS-ML, we generated iPS-ML expressing scFv specific for human HER2/neu. The scFv was linked to the trans-membrane and cytoplasmic domains of FcγRI. Expression of anti-HER2 scFv made the iPS-ML able to reduce the growth of HER2-expressing gastric cancer cells, NUGC-4, in vitro (Fig. 2). However, iPS-ML/anti-HER2 promoted rather than inhibited the progression of cancer *in vivo* (Fig. 4).

Local cytokine milieu is the critical factor determining whether TAM exert pro- or anti-tumor activity [1]. It is considered that TAM are affected by the cancer microenvironment and polarized to M2 phenotype in most cases. In similar to the naturally occurring macrophages, iPS-ML or iPS-ML/anti-HER2 may have been affected by the environment to exert pro-cancer activity after infiltration into the cancer tissues.

To make iPS-ML able to overcome the influence of tumor environment and to efficiently attack cancer cells, we introduced expression vectors for anti-cancer molecules into iPS-ML. We searched for factors that could enhance the anti-cancer effect of iPS-ML/anti-HER2. NUGC-4 cells are sensitive to IFN-α, IFN-β, IFN-γ, and TNF-α?as adding these factors into cultures induced apoptosis and reduced growth of NUGC-4 cells (Fig. S2). We observed that iPS-ML/anti-HER2 expressing IFN-α, IFN-β, or TRAIL inhibited NUGC-4 cell growth more potently than iPS-ML/anti-HER2 *in vitro* (Fig. 4). However, iPS-ML/anti-HER2/IFN-α and iPS-ML/anti-HER2/TRAIL exhibited no effect on the growth of peritoneally implanted NUGC-4 gastric cancer *in vivo*. On the other hand, iPS-ML/IFN-β irrespective of expression of anti-HER2, significantly inhibited NUGC-4 cancer progression (Fig. 5).

As described above, despite that both transfectant iPS-ML-derived IFN-α and IFN-β showed anti-cancer effect *in vitro*, only iPS-ML/IFN-β exhibited anti-cancer effect in the xenograft model. One possible reason for the superior *in vivo* effect of iPS-ML/IFN-β as compared with iPS-ML/IFN-αmay be the difference in tissue affinity between IFN-α and IFN-β. IFN-β is more lipophilic than IFN-α and thus possesses greater tissue affinity [34]. IFN-β produced by iPS-ML in the cancer tissues may have probably retained in situ longer than IFN-α, resulting in higher local concentration and exhibition of more potent anti-cancer effect.

Gastric cancer is one of the major types of malignancy worldwide and the second most frequent cause of cancer-related mortality [35]. Peritoneal dissemination is the most difficult type of metastasis of gastric cancer to treat [36]. At present, there is no standard therapy against peritoneally disseminated gastric cancer [37]. In addition to NUGC-4 gastric cancer, iPS-ML/IFN-β inhibited the growth of MIAPaCa-2 pancreatic cancer in the xenograft model (Fig. 6). Pancreatic cancer is also one of the most common causes of cancer-related mortality in the world [38]. Pancreatic cancer has a poor prognosis, with the overall 5-year survival rate at less than 10%. More than half of the patients diagnosed with pancreatic cancer are not suitable for surgical resection, and their mean survival time is less than 1 year. Therefore, development of therapies to treat these intractable types of cancers is urgently needed. The present study suggests the possible clinical application of iPS-ML producing anti-cancer factors to treat cancers for which no standard therapy has been established.

To achieve clinical effect in treating cancer patients by macrophage therapy, repetitive administration of a large number of cells may be necessary. Since iPS-ML proliferate for at least several months, sufficient iPS-ML numbers can readily be supplied. However, the proliferative capacity of iPS-ML may cause concern, as leukemia development from iPS-ML is possible if autologous iPS-ML are administered into patients.

To avoid the risk of leukemia, we plan to use allogeneic iPS-ML deficient in the TAP (transporter associated with antigen presentation) molecule in future clinical applications. TAP plays key role in antigen presentation by HLA class I. Antigenic peptides to be presented by HLA class I are transported from the cytoplasm to the lumen of the endoplasmic reticulum by TAP [39]. In TAP-deficient cells, cell surface expression levels of HLA class I is very low. More importantly, TAP deficiency greatly reduces the complexity of peptides presented on the HLA class I molecules. Thus, TAP-deficient allogeneic iPS-ML will evade recognition by most of allo-reactive CD8^+^ T cells (the major immune effector cells mediating acute rejection) upon cell transfer into allogeneic recipients [40]–[42].

Nevertheless, the allo-reactive CD8^+^ T cells recognizing HLA class I-bound peptides presented via the TAP-independent pathway (mainly derived from signal peptides) [43] may eventually eliminate the transferred allogeneic iPS-ML. Additionally, since the HLA class II molecules are intact even in TAP-deficient iPS-ML, allo-reactive CD4^+^ T cell may also attack the cells. Collectively, we predict that the injected iPS-ML will survive in the recipients for several days to exert the anti-cancer effect, but then will be completely eliminated by the recipient’s immune system. Thus, we consider that therapy with allogeneic TAP-deficient iPS-ML is effective and safe.

In summary, iPS-ML accumulated and infiltrated into the tumor tissues upon i.p. injection into SCID mice bearing peritoneally implanted cancer. iPS-ML expressing IFN-β inhibited the growth of MIAPaCa-2 pancreatic cancer as well as NUGC-4 gastric cancer in xeno-graft models. Although we reasoned that adoptive anti-cancer cell therapy with TAP-deficient allogeneic iPS-ML is effective and safe, further preclinical study is necessary.

## Supporting Information

Figure S1 **No effect of iPS-ML/anti-HER2 on the growth of peritoneally disseminated NUGC-4 cells.** Luciferase-expressing NUGC-4 cells (5×10^6^ cells/mouse) were injected into the peritoneal cavity of SCID mice. After 3 days, mice were subjected to bioluminescence analysis to detect cancer cells in the peritoneal cavity. Mice exhibiting evident luminescence signals were randomly divided into control (n = 6) and therapy (n = 3) groups. Mice in the therapy group were injected i.p. with iPS-ML/anti-HER2 (2×10^7 ^cells/mouse each day) daily from days 4–8. On day 10 or 11, the mice were analyzed again to analyze tumor growth. **A**. The luminescence images on day 3 and day 10/11 are shown. **B**. For each mouse, fold change in luminescence signal from day 3 to day 10/11 was calculated. The mean + SD of fold change for each group is shown.
(TIF)

Figure S2 **Effect of TNF-α and IFNs to induce apoptosis of NUGC-4 cells. A.** NUGC-4 cells were cultured in a 24-well culture plate (2.5×10^4^ cells/well in 1 mL) in the presence or absence of TNF-αIFN-α, IFN-β, or IFN-γ all 10 ng/mL). After 3 days, cells were recovered, stained with FITC-labeled Annexin-V, and analyzed on a flow cytometer to detect apoptotic cells. The numbers in the figures indicate the percentage of cells positively stained with annexin-V. **B.** Luciferase-expressing NUGC-4 cells (5×10^3 ^cells/well) were cultured in a 96-well culture plate in the presence or absence of TNF-α, IFN-α, IFN-β, or IFN-γ (10 ng/mL). The number of live NUGC-4 cells was measured by luciferase activity after a 3-day culture. The data are indicated as the mean ± SD of triplicate assays.
(TIF)

Figure S3 **Generation of iPS-ML expressing IFNs, TNF-α, or TRAIL along with anti-HER2 scFv. A.** iPS-ML transduced with lentivirus vector for IFNs, TNF-α, or FAS-ligand were cultured (2×10^5 ^cells/well in 200 µL) in 96-well culture plates. After 24 hours, culture supernatant was collected, and the concentration of each cytokine was measured by ELISA. Culture medium alone and iPS-ML/anti-HER2 supernatant were also analyzed as controls. **B.** Cell-surface expression of TRAIL on iPS-ML transduced with the TRAIL expression vector was examined by flow cytometric analysis. The staining profiles of the specific mAb (thick line) and an isotype-matched control mAb (grey area) are shown.
(TIF)

Figure S4 **Effect of iPS-ML/IFN-β and recombinant IFN-β on peritoneally disseminated NUGC-4 cells.** Luciferase–expressing NUGC-4 cells were injected i.p. into SCID mice (5×10^6 ^cells/mouse). On day 3, the mice were subjected to the luminescence imaging analysis. Mice were injected on day 4, 6, and 8 with iPS-ML (2×10^7^ cells, n = 5), iPS-ML/anti-HER2 (2×10^7^ cells, n = 5), iPS-ML/IFN-β (2×10^7^ cells, n = 5), 200 ng of recombinant IFN-β (n = 5), or 400 ng of recombinant IFN-β (n = 4). As a control, 5 mice were left untreated. All mice were subjected to bioluminescence analysis again on day 11. **A.** The luminescence images are shown. **B.** For each mouse, fold change in luminescence signal from day 3 to day 11 was calculated. The mean + SD of fold change for each group is shown.
(TIF)

Figure S5 **Effect of IFN-β to induce apoptosis of MIAPaCa-2 cells in vitro. A.** MIAPaCa-2 cells were cultured in a 24-well culture plate (2.5×10^4^ cells/well in 1 mL) in the presence or absence of IFN-β (10 ng/mL). After 3 days, cells were recovered, stained with FITC-labeled Annexin-V, and analyzed on a flow cytometer to detect apoptotic cells. The numbers in the figures indicate the percentage of cells positively stained with annexin-V. **B.** Luciferase-expressing NUGC-4 cells (5×10^3^ cells/well) were cultured in a 96-well culture plate in the presence or absence of IFN-β (10 ng/mL). The number of live NUGC-4 cells was measured by luciferase activity after a 3-day culture. The data are indicated as mean + SD of triplicate assays.
(TIF)
