# Supplementary figures and images for: Therapeutic Effect of Human iPS-Cell–Derived Myeloid Cells Expressing IFN-β against Peritoneally Disseminated Cancer in Xenograft Models
Source: PLoS One. 2013 Jun 24;8(6):e67567. doi: 10.1371/journal.pone.0067567 (PMC3691167; doi:10.1371/journal.pone.0067567)

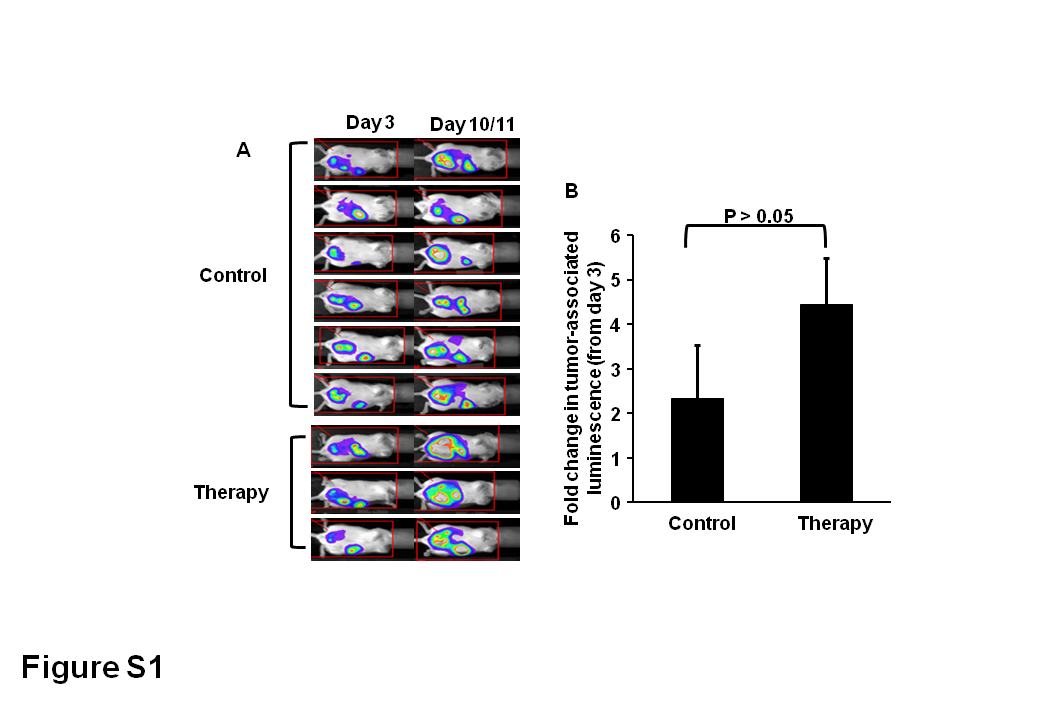

Supplement: Figure S1 — No effect of iPS-ML/anti-HER2 on the growth of peritoneally disseminated NUGC-4 cells. Luciferase-expressing NUGC-4 cells (5×10^6^ cells/mouse) were injected into the peritoneal cavity of SCID mice. After 3 days, mice were subjected to bioluminescence analysis to detect cancer cells in the peritoneal cavity. Mice exhibiting evident luminescence signals were randomly divided into control (n = 6) and therapy (n = 3) groups. Mice in the therapy group were injected i.p. with iPS-ML/anti-HER2 (2×10^7^ cells/mouse each day) daily from days 4–8. On day 10 or 11, the mice were analyzed again to analyze tumor growth. A. The luminescence images on day 3 and day 10/11 are shown. B. For each mouse, fold change in luminescence signal from day 3 to day 10/11 was calculated. The mean + SD of fold change for each group is shown. (TIF) [file pone.0067567.s001.tif]

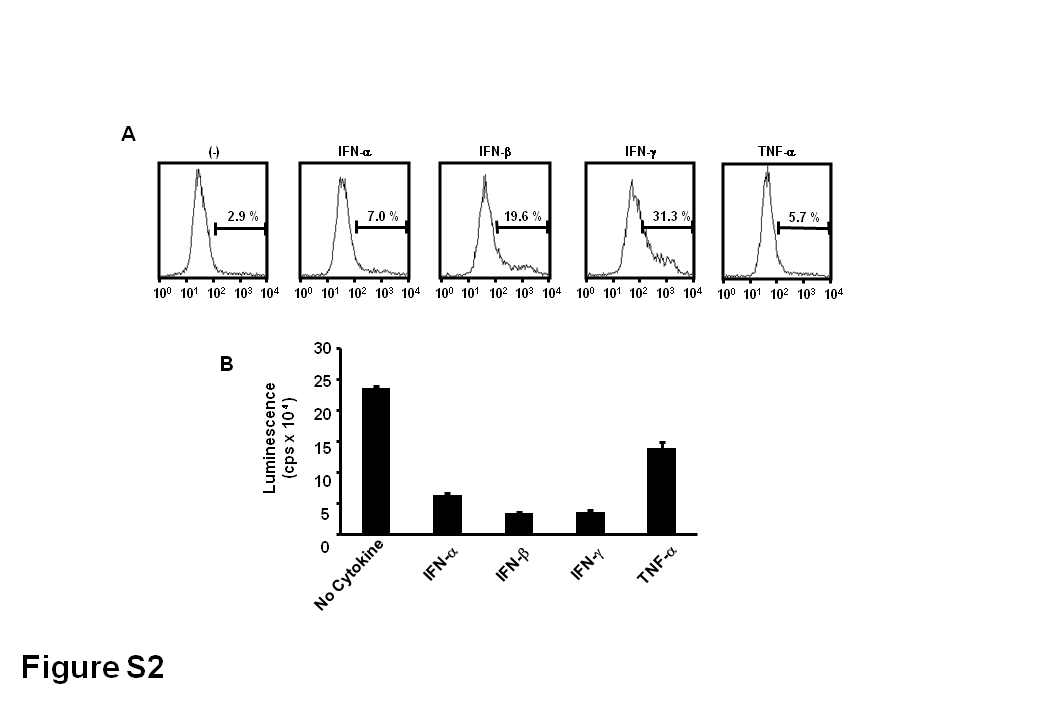

Supplement: Figure S2 — Effect of TNF-α and IFNs to induce apoptosis of NUGC-4 cells. A. NUGC-4 cells were cultured in a 24-well culture plate (2.5×10^4^ cells/well in 1 mL) in the presence or absence of TNF-αIFN-α, IFN-β, or IFN-γ all 10 ng/mL). After 3 days, cells were recovered, stained with FITC-labeled Annexin-V, and analyzed on a flow cytometer to detect apoptotic cells. The numbers in the figures indicate the percentage of cells positively stained with annexin-V. B. Luciferase-expressing NUGC-4 cells (5×10^3^ cells/well) were cultured in a 96-well culture plate in the presence or absence of TNF-α, IFN-α, IFN-β, or IFN-γ (10 ng/mL). The number of live NUGC-4 cells was measured by luciferase activity after a 3-day culture. The data are indicated as the mean ± SD of triplicate assays. (TIF) [file pone.0067567.s002.tif]

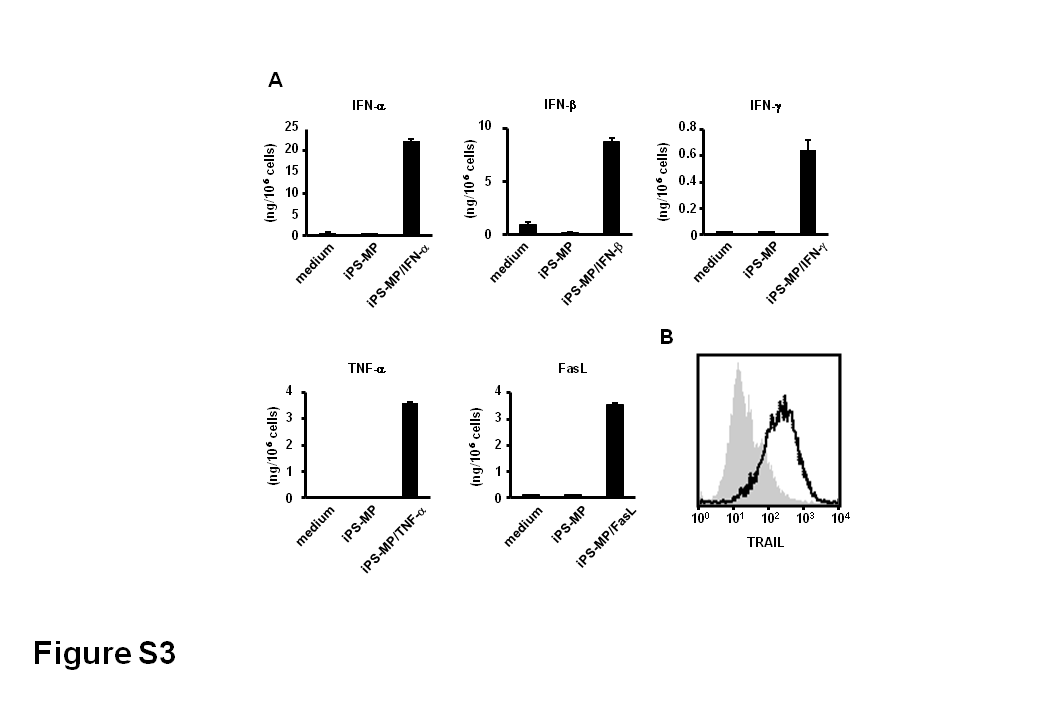

Supplement: Figure S3 — Generation of iPS-ML expressing IFNs, TNF-α, or TRAIL along with anti-HER2 scFv. A. iPS-ML transduced with lentivirus vector for IFNs, TNF-α, or FAS-ligand were cultured (2×10^5^ cells/well in 200 µL) in 96-well culture plates. After 24 hours, culture supernatant was collected, and the concentration of each cytokine was measured by ELISA. Culture medium alone and iPS-ML/anti-HER2 supernatant were also analyzed as controls. B. Cell-surface expression of TRAIL on iPS-ML transduced with the TRAIL expression vector was examined by flow cytometric analysis. The staining profiles of the specific mAb (thick line) and an isotype-matched control mAb (grey area) are shown. (TIF) [file pone.0067567.s003.tif]

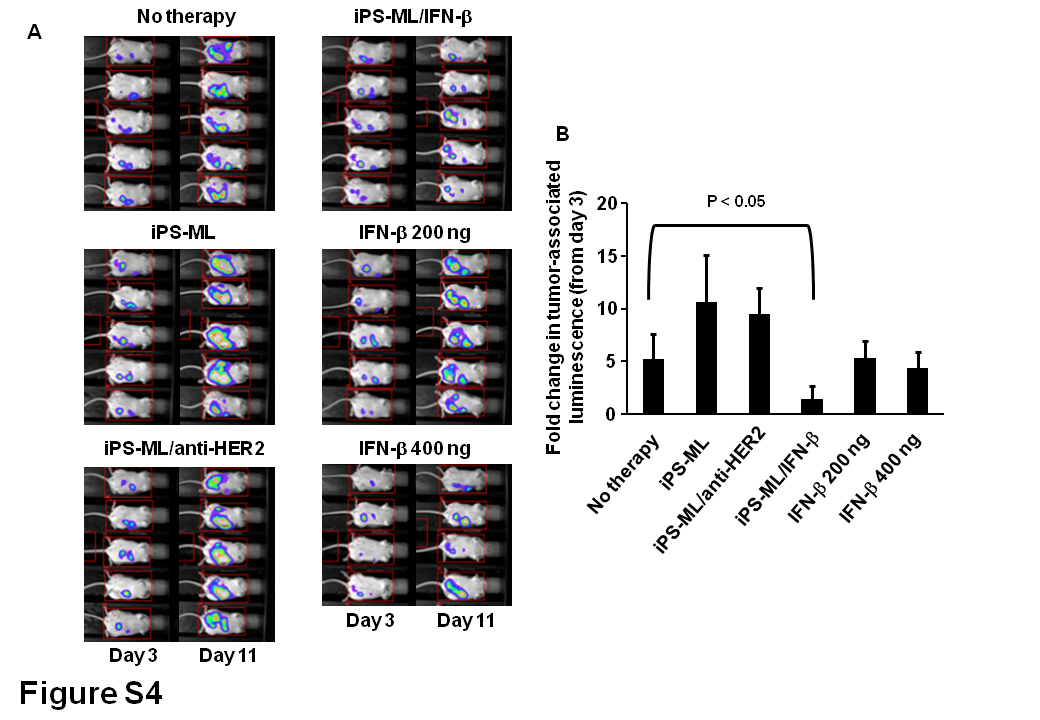

Supplement: Figure S4 — Effect of iPS-ML/IFN-β and recombinant IFN-β on peritoneally disseminated NUGC-4 cells. Luciferase–expressing NUGC-4 cells were injected i.p. into SCID mice (5×10^6^ cells/mouse). On day 3, the mice were subjected to the luminescence imaging analysis. Mice were injected on day 4, 6, and 8 with iPS-ML (2×10^7^ cells, n = 5), iPS-ML/anti-HER2 (2×10^7^ cells, n = 5), iPS-ML/IFN-β (2×10^7^ cells, n = 5), 200 ng of recombinant IFN-β (n = 5), or 400 ng of recombinant IFN-β (n = 4). As a control, 5 mice were left untreated. All mice were subjected to bioluminescence analysis again on day 11. A. The luminescence images are shown. B. For each mouse, fold change in luminescence signal from day 3 to day 11 was calculated. The mean + SD of fold change for each group is shown. (TIF) [file pone.0067567.s004.tif]

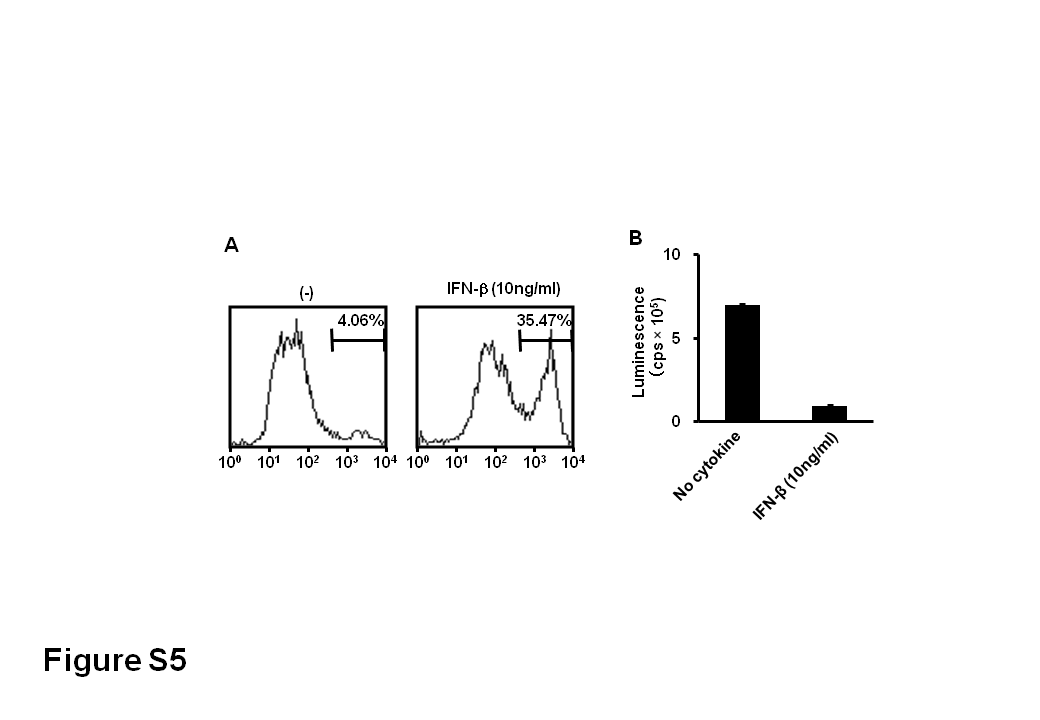

Supplement: Figure S5 — Effect of IFN-β to induce apoptosis of MIAPaCa-2 cells in vitro. A. MIAPaCa-2 cells were cultured in a 24-well culture plate (2.5×10^4^ cells/well in 1 mL) in the presence or absence of IFN-β (10 ng/mL). After 3 days, cells were recovered, stained with FITC-labeled Annexin-V, and analyzed on a flow cytometer to detect apoptotic cells. The numbers in the figures indicate the percentage of cells positively stained with annexin-V. B. Luciferase-expressing NUGC-4 cells (5×10^3^ cells/well) were cultured in a 96-well culture plate in the presence or absence of IFN-β (10 ng/mL). The number of live NUGC-4 cells was measured by luciferase activity after a 3-day culture. The data are indicated as mean + SD of triplicate assays. (TIF) [file pone.0067567.s005.tif]
